# Analysis of the Interaction between *Pisum sativum* L. and *Rhizobium laguerreae* Strains Nodulating This Legume in Northwest Spain

**DOI:** 10.3390/plants9121755

**Published:** 2020-12-11

**Authors:** José David Flores-Félix, Lorena Carro, Eugenia Cerda-Castillo, Andrea Squartini, Raúl Rivas, Encarna Velázquez

**Affiliations:** 1 Departamento de Microbiologíay Genética, Universidad de Salamanca, 37007 Salamanca, Spain; jdflores@usal.es (J.D.F.-F.); lcg@usal.es (L.C.); eugenia.castillo@ct.unanleon.edu.ni (E.C.-C.); raulrg@usal.es (R.R.); 2 Department of Agronomy, Food, Natural Resources, Animals and Environment, University of Padova, 35020 Legnaro, Italy; squart@unipd.it; 3 Instituto Hispanoluso de Investigaciones Agrarias, 37007 Salamanca, Spain; 4 Unidad Asociada USAL-IRNASA, 37007 Salamanca, Spain

**Keywords:** pea, *Pisum sativum*, *Rhizobium laguerreae*, symbiosis, quorum sensing, nodulation effectiveness, nitrogen fixation

## Abstract

*Pisum sativum* L. (pea) is one of the most cultivated grain legumes in European countries due to the high protein content of its seeds. Nevertheless, the rhizobial microsymbionts of this legume have been scarcely studied in these countries. In this work, we analyzed the rhizobial strains nodulating the pea in a region from Northwestern Spain, where this legume is widely cultivated. The isolated strains were genetically diverse, and the phylogenetic analysis of core and symbiotic genes showed that these strains belong to different clusters related to *R. laguerreae* sv. viciae. Representative strains of these clusters were able to produce cellulose and cellulases, which are two key molecules in the legume infection process. They formed biofilms and produced acyl-homoserine lactones (AHLs), which are involved in the quorum sensing regulation process. They also exhibited several plant growth promotion mechanisms, including phosphate solubilization, siderophore, and indole acetic acid production and symbiotic atmospheric nitrogen fixation. All strains showed high symbiotic efficiency on pea plants, indicating that strains of *R. laguerreae* sv. viciae are promising candidates for the biofertilization of this legume worldwide.

## 1. Introduction

*Pisum sativum* L. (pea) is one of the most cultivated legumes worldwide due to the high protein content and quality of its seeds [1], with a cultivated area higher than 8 million ha and a production of over 16 million tonnes per year, about 44% of which corresponds to Europe [2]. Spain is within the top 10 countries producing dry peas, with a production in 2018 of 262,567 tonnes of dry peas and 109,270 tonnes of green peas. Around 30% were produced in the Castilla y León region, the Valladolid province being the highest producer of dry peas within this region with 39,980 tonnes (52% of the total production) according to the Spanish Ministry of Agriculture, Fishing and Food (https://www.mapa.gob.es/es/estadistica/temas/publicaciones/anuario-de-estadistica/2019/default.aspx) [3]. 

Like other legumes, *P. sativum* establishes symbiosis with fast-growing rhizobia, particularly with species within the genus *Rhizobium*, which encompasses several species whose type strains were originally isolated from *P. sativum* nodules, such as *Rhizobium leguminosarum* [4], *Rhizobium pisi* [5], *Rhizobium indicum* [6], and *Rhizobium ruizarguesonis* [7]. Of these, only the strains from *R. ruizarguesonis* have been isolated in European countries such as Italy and Germany [7]. In the case of *Rhizobium anhuiense*, although the type strain was isolated from *Vicia faba* nodules, several strains isolated from *P. sativum* nodules were included in its original description [8].

Although the diversity of rhizobia nodulating *P. sativum* in some countries has been analyzed in the last decade [9,10,11,12,13,14,15], the endosymbionts of this legume have scarcely been studied in Spain, despite the economic importance of pea crops in this country. Some strains isolated from pea nodules in Central Spain were identified as *R. leguminosarum* on the basis of *rrs* gene (16S rRNA) sequence analysis [10] and other strains isolated in Northwestern Spain were identified as *R. leguminosarum* or *R. laguerreae* by MALDI-TOF MS analysis [12]. Of these strains, the nodulation genes have only been analyzed for the *R. leguminosarum* strains isolated in Central Spain, which belong to the symbiovar viciae [10]. The type strains of the previously mentioned species, *R. leguminosarum*, *R. pisi*, *R. anhuiense,* and *R. ruizarguesonis* also belong to this symbiovar. Moreover, strains of this symbiovar linked to *R. leguminosarum* and *R. laguerreae* have been found in *P. sativum* nodules in Turkey [11] and Morocco [15].

The interaction between *P. sativum* and different strains of the species *R. leguminosarum* has been widely studied since the proposal of this bacterial name for pea nodulating bacteria [4]. However, the interaction between this legume and other more recently described *Rhizobium* species has been scarcely studied. Therefore, the objectives of this work were (i) an analysis of the diversity of *R. laguerreae* strains nodulating *P. sativum* in a soil from Northwest Spain, (ii) an analysis of their phylogenetic relationships with strains of *R. laguerreae* isolated in other geographical locations and from other legume hosts on the basis of core and symbiotic genes, (iii) a study of the ability to form biofilms in abiotic surfaces, of the production of molecules involved in the colonization of *P. sativum*, of quorum sensing, and of plant growth promotion, and (iv) an evaluation of the effectiveness of these strains on pea plants.

## 2. Results and Discussion

### 2.1. RAPD Fingerprinting

**Figure 1:**
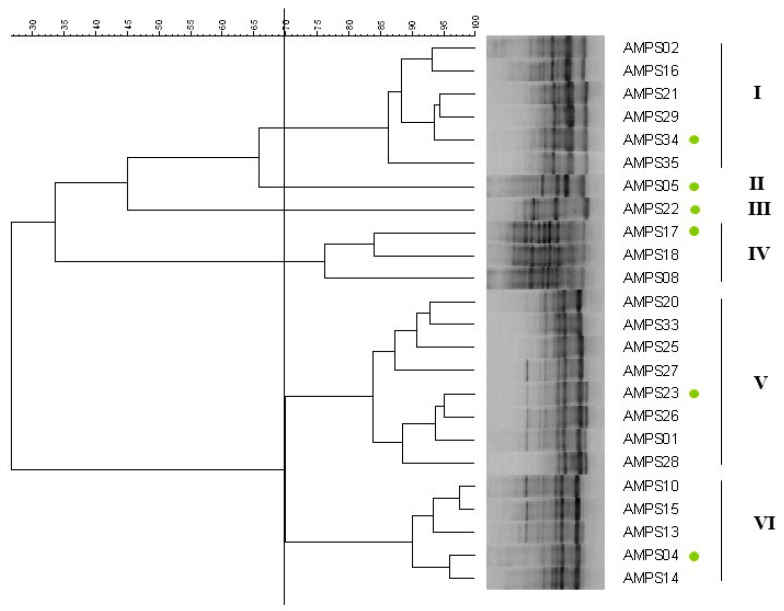
Dendrogram obtained for the strains of this study using Pearson’s coefficient and UPGMA (Unweighted Pair Group Method with Arithmetic Mean) analysis of the RAPD profiles. Filled green circles indicated the strains selected from different RAPD groups for the remaining analysis of this study.

The RAPD fingerprinting has been widely used for the diversity analysis of fast-growing rhizobia, particularly those isolated from different legumes of the *Vicia* cross-nodulation group [16,17,18,19,20]. Therefore, we analyzed the RAPD patterns displayed by 24 rhizobial strains isolated from *P. sativum* nodules [12]. The results of this analysis showed that the strains from this study were genetically diverse and distributed into six groups with similarity values lower than 70% (Figure 1). A representative strain from each one of these groups was subjected to gene sequencing.

### 2.2. Analysis of the rrs Gene 

**Figure 2:**
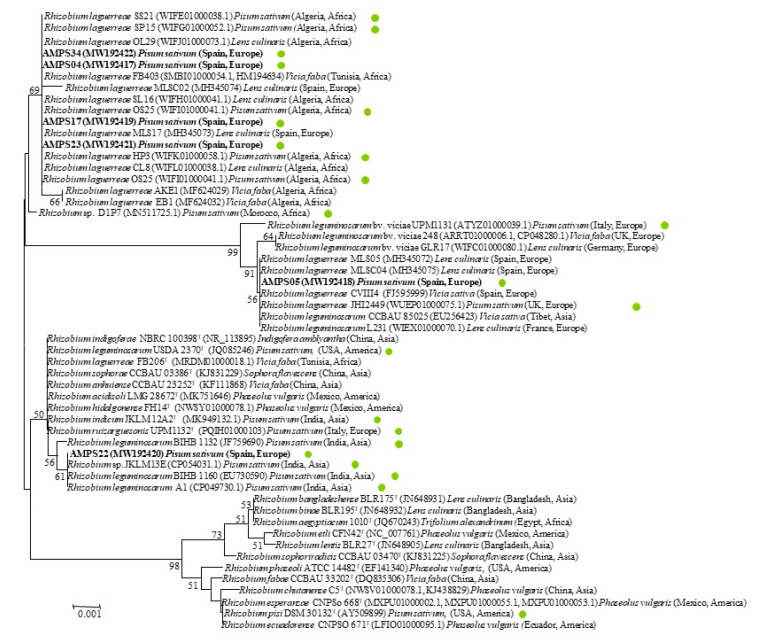
Neighbor-joining unrooted phylogenetic tree based on *rrs* gene sequences (1340 nt) showing the taxonomic location of representative strains from different groups of RAPD within the genus *Rhizobium*. Bootstrap values calculated for 1000 replications are indicated. Bar: 1 nt substitution per 1000 nt. Filled green circles indicated the strains isolated from *Pisum sativum* worldwide.

The *rrs* gene has been sequenced in many strains nodulating *Pisum* and other legumes from the same cross-inoculation group, such as *Vicia*, *Lens*, and *Lathyrus*, some of them isolated in different Spanish regions [20,21]. These strains belong to the phylogenetic group of *R. leguminosarum*, which contains several species whose type strains have identical *rrs* genes (Figure 2). However, slight differences have been found in the *rrs* gene sequences of other strains as in the case of strains belonging to the species *R. laguerreae* in which some strains harbored an insert of about 70 bp at the beginning of their *rrs* genes [21]. The results of this study showed that the analyzed strains belong to different phylogenetic clusters, each of which also contain other strains isolated from *Pisum*, *Vicia*, or *Lens* in different countries and continents (Figure 2). 

All strains from this study presented differences in their *rrs* genes with respect to those of the type strains of the species from the *R. leguminosarum* group. Concretely, the *rrs* genes of the strains AMPS34, AMPS17, AMPS23, and AMPS04, representative of RAPD groups I, IV, V, and VI, respectively, have two different nucleotides with respect to these strains (positions 942 and 955, taking the *rrs* gene sequence of *R. laguerreae* FB206^T^ with accession number JN558651 as a reference). The *rrs* gene sequences of these strains were identical to those of several strains of *R. laguerreae* isolated from nodules of *Vicia* and *Lens* in different countries, highlighting the strain MLS17 isolated from nodules of *L. culinaris* in Spain [21] and the strain FB403 included in the original description of *R. laguerreae*, which was isolated from *V. faba* nodules in Tunisia [22]. The *rrs* gene sequence of strain AMPS05, representative of RAPD type II, presented four different nucleotides (positions 67, 69, 942, and 955) and contains an intervening sequence (IVS) of about 70 bp located at the beginning of this gene between nucleotides 70 and 143. The *rrs* gene sequence and the IVS of strain AMPS05 were identical to those found in strains *R. laguerreae* MLS05 and MLSC04, which nodulated *L. culinaris* in Spain [21], in strain CVIII4, included in the original description of *R. laguerreae*, which nodulated *V. sativa* in Spain [22], and in other strains isolated from nodules of *Pisum*, *Vicia*, and *Lens* in different countries (Figure 2). Finally, the *rrs* gene sequence of strain AMPS22, representative of RAPD group III, has a different nucleotide at the position 1070, and it was identical to those of two strains isolated from nodules of *P. sativum* in India and a strain isolated from the same host in Russia (Figure 2).

It is remarkable that, although most strains with *rrs* gene sequences identical to those of our strains have been assigned to the species *R. laguerreae*, some of them are named *R. leguminosarum* or *Rhizobium* sp. The elucidation of the taxonomic affiliation of these strains cannot be based on the *rrs* gene sequences since they are identical in several type strains of different species, namely *R. leguminosarum*, *R. laguerreae, R. anhuiense, R. sophorae, R. acidisoli, R. hidalgonense, R. ruizarguesonis*, and *R. indicum* (Figure 2). It should be based on the analysis of housekeeping genes, such as *recA* and *atpD*, which allow us to differentiate among species with identical *rrs* gene sequences. In this study, we revised the affiliation of some of these strains for which the *recA* and *atpD* gene sequences are currently available.

### 2.3. Analysis of recA and atpD Genes

**Figure 3:**
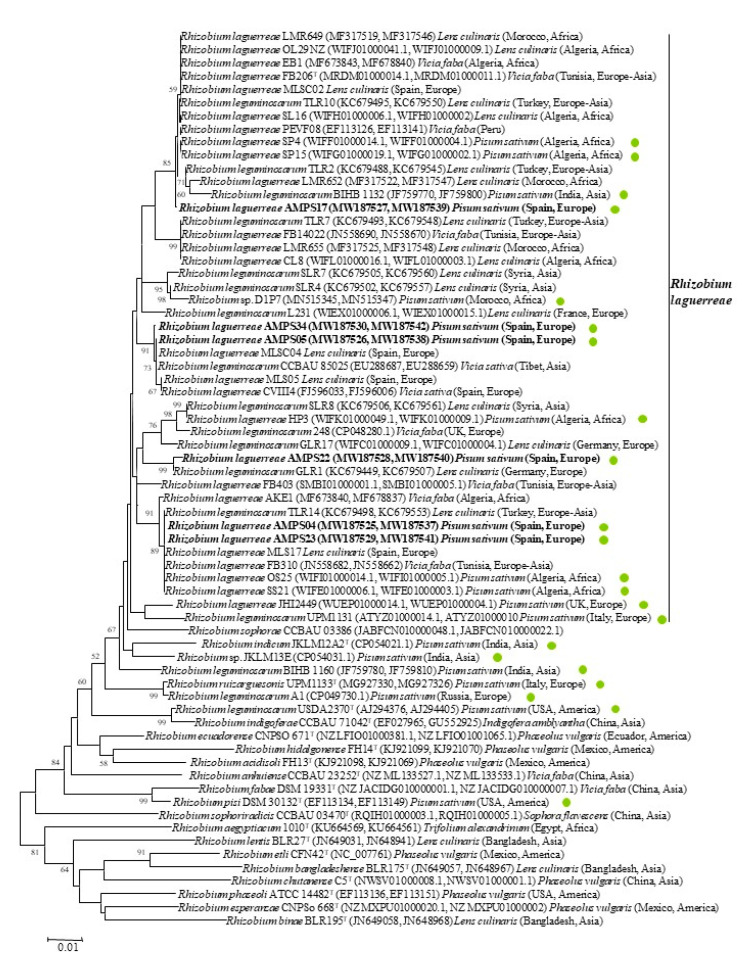
Neighbor-joining unrooted phylogenetic tree based on *recA* and *atpD* concatenated gene sequences (728 nt) showing the position of representative strains from each group within genus *Rhizobium*. Bootstrap values calculated for 1000 replications are indicated. Bar: 1 nt substitution per 100 nt. Filled green circles indicated the strains isolated from *Pisum sativum* worldwide.

The *atpD* and *recA* genes were the first housekeeping genes applied to species differentiation within the family *Rhizobiaceae* [23] and therefore have been sequenced for the type strains of all *Rhizobium* species described to date, allowing for the assignment of new isolates to these species. In addition, their sequences are available for many other strains, particularly for those nodulating *P. sativum* and other legumes from the *Vicia* cross-inoculation group in different geographical locations (Figure 3).

The results of the analysis of *recA* and *atpD* genes showed that the strains analyzed in this work present similarity values higher than 96% and 98%, respectively, with respect to the type strain of *R. laguerreae* (Table 1). These values showed that all of these strains belong to the species *R. laguerreae*, even though they matched with score values lower than 2.3 with the type strain of this species after MALDI-TOF MS analysis [12]. The phylogenetic analysis of the concatenated *recA* and *atpD* genes showed that the analyzed strains belong to different clusters, some of which also contain strains included in the official description of the species *R. laguerreae* [22]. The strain AMPS17 belongs to the same phylogenetic cluster of the type strain of this species FB206^T^, strains AMPS05 and AMPS34 belong to that of the strain CVIII4, and strains AMPS04 and AMPS23 belong to that of the strain FB310 (Figure 3). 

In the phylogenetic analysis of the *recA* and *atpD* genes, we also included strains closely related to those from this study, when the sequences of these two genes are available at GenBank (Figure 3). Several of these strains isolated from nodules of *Pisum, Vicia*, and *Lens*, including those strains carrying an IVS in their *rrs* gene, have been named *Rhizobium leguminosarum*, but the analysis of their *recA* and *atpD* genes placed them within the species *R. laguerreae* (Figure 3). This analysis confirmed the phylogenetic complexity of this species, which was already shown at the time of its description, since it encompassed strains phylogenetically divergent [22] and led Flores-Félix et al. [21] to point out the existence of several genospecies within *R. laguerreae* after the analysis of several strains isolated from nodules of different legumes [21]. Nevertheless, in the present work, where we analyzed more strains from the phylogenetic group of *R. laguerreae*, we observed a high complexity within this species, indicating that it is difficult to establish reasonable limits for differentiating genospecies (or subspecies) and that it will be necessary to do so based on the analysis of whole genomes.

The strains nodulating *P. sativum* analyzed in this study fall within four clusters obtained after the *recA* and *atpD* gene analysis that also contain other strains isolated in different countries from nodules of *Lens*, *Vicia*, and *Pisum* (Figure 3). Strain AMPS17 and strains AMPS04 and AMPS23 belong to two clusters, together with closely related strains, most of which are already named *R. laguerreae* and have been isolated in different countries of Asia, Africa, Europe, and America from nodules of *L. culinaris* (Turkey, Algeria, and Morocco), *V. faba* (Tunisia, Algeria, and Peru), and *P. sativum* (Algeria and India) [9,15,17,22,24,25]. Some of these strains were named *R. leguminosarum* at the time of their publication because the species *R. laguerreae* was described later [9,25]. Strains AMPS05 and AMPS34 belong to a cluster containing strains isolated from *Vicia sativa* in Asia and Europe [21,22,26]. Finally, the strain AMPS22 was closely related to a strain isolated by Rashid et al. [25] from nodules of *L. culinaris* in Germany (Figure 3). It is remarkable that, from the strains isolated from *P. sativum* nodules whose *rrs* sequences were almost identical to that of the strain AMPS22, only the strain BIHB1132 belongs to *R. laguerreae*. The strains A1 and JKLM13E, were related to the type strains of *R. ruizarguesonis* and *R. indicum*, respectively, and the strain BIHB1160 formed and independent lineage (Figure 3). In the case of the strains carrying the IVS and whose *rrs* gene sequences were identical, we also found important divergences for *recA* and *atpD* genes, particularly in the case of the strain JHI2449, which showed 96.3% and 98.2% similarity, respectively, with respect to the type strain of *R. laguerreae*, these values being within the limit for species differentiation. Nevertheless, we also found the opposite case, since strains with some differences in the *rrs* gene, such as the strain AMPS17, were closely related to the type strain of *R. laguerreae* in the *recA* and *atpD* gene analysis. These results confirmed again that the *rrs* gene is not a suitable marker for differentiation of closely related species, other core protein-coding genes being more adequate for species delineation.

**Table 1:** Characteristics of selected strains isolated from *Pisum sativum* root nodules.

Strain	RAPD Group	*recA* Gene * Similarity (%)	*atpD* Gene * Similarity (%)	Cellulases Production	Cellulose Production	AHL Production ^§^	IAA (mg/L)	Siderophore Production ^¥^	Phosphate Solubilization ^ƒ^
AMPS04	VI	96.7	100	w	w	1:125	0.082	4	0.00
AMPS05	II	96.8	99.4	w	+	1:125	0.081	5	0.00
AMPS17	IV	99.8	100	w	+	1:3125	0.040	4	0.00
AMPS22	III	96.5	98.1	+	+	1:3125	0.023	1	0.00
AMPS23	V	96.7	100	+	+	1:25	0.023	1	1.33
AMPS34	I	97.6	99.5	+	w	1.25	0.079	7	1.17

*: this value was calculated with respect to the type strain of *R. laguerreae* FB206^T^. w: weakly positive, +: positive. ^§^: last dilution in which AHL was detected (following the methodology of Trovato et al. [27]). ^¥^: halo size (mm) around colony. ^ƒ^: ratio between the diameter of the halo around the colony with respect to colony size [28].

### 2.4. Analysis of the nodC Gene 

**Figure 4:**
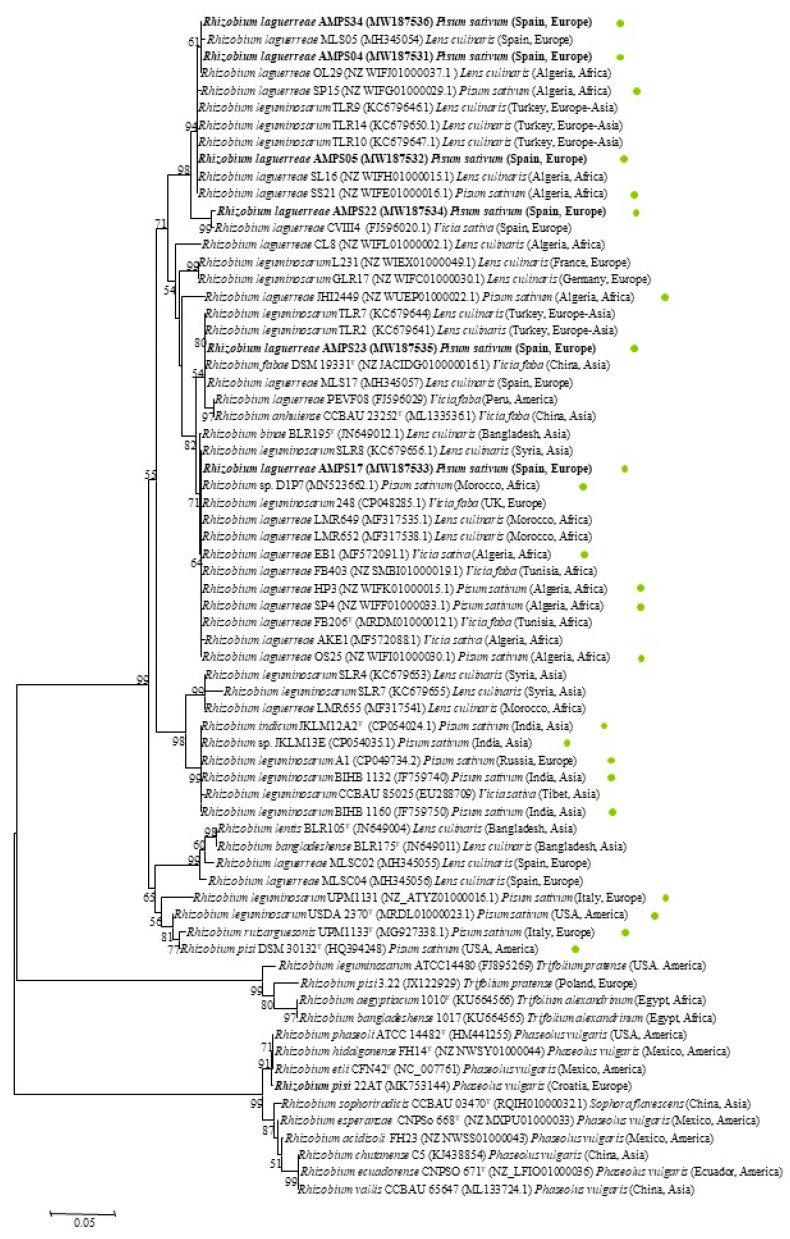
Neighbor-joining unrooted phylogenetic tree based on *nodC* gene sequences (370 nt) showing the position of representative strains from each group within the symbiovars viciae and phaseoli. Bootstrap values calculated for 1000 replications are indicated. Bar: 5 nt substitution per 100 nt. Filled green circles indicated the strains isolated from *Pisum sativum* worldwide.

The nodulation genes are responsible for the legume nodule formation, and among them, the *nodC* gene is the most common phylogenetic marker to define rhizobial symbiovars [29,30]. The symbiovar concept (formerly biovar) is related to both the rhizobial host range and the legume promiscuity degree and hence to the old cross-inoculation group concept [30]. In this way, a promiscuous legume can be nodulated by various symbiovars, and a restrictive legume can only be nodulated by one symbiovar [30]. The legumes from the *Vicia* cross-inoculation group, which includes *Vicia*, *Pisum*, *Lens*, and *Lathyrus*, are considered restrictive hosts for nodulation [31], nodulated by strains of the symbiovar viciae [32]. It is remarkable that a single species can contain several symbiovars; the first reported case was the species *R. leguminosarum*, which contains three symbiovars, two of which nodulate restrictive hosts such as *Vicia*, *Pisum*, *Lens*, *Lathyrus* (viciae), and *Trifolium* (trifolii) and one of which nodulates the very promiscuous host *Phaseolus* (phaseoli) [32]. These three symbiovars are clearly separated through *nodC* gene analysis [33,34], and for this reason it has been analyzed in many strains isolated from nodules of legumes from the *Vicia* cross-inoculation group in different geographical locations (Figure 4). 

The analysis of the *nodC* gene showed that, as expected, the closest related strains to those from this study are those of the symbiovar viciae, with similarity values higher than 92%. Nevertheless, the phylogenetic analysis of the *nodC* gene showed that these strains belong to two different clusters (Figure 4). Strains AMPS04, AMPS34, AMPS05, and AMPS22 belong to a cluster that encompasses strains isolated from nodules of *Pisum*, *Vicia*, and *Lens* in several countries in Europe and Africa, and strains AMPS17 and AMPS23 belong to a wide cluster encompassing strains nodulating the same legumes in different countries in Europe, America, Asia, and Africa. These results confirmed the existence of an important genetic variability within the symbiovar viciae worldwide, with similarity values lower than 95% in the *nodC* gene. This value is within the range of internal similarity of most defined symbiovars, but it is lower than the values reported for the strains of the novel symbiovar aegeanense recently described within the genus *Ensifer*, which showed more than 95% similarity with respect to the symbiovar fredii [35]. 

### 2.5. Biofilm Formation and Quorum Sensing

**Figure 5:**
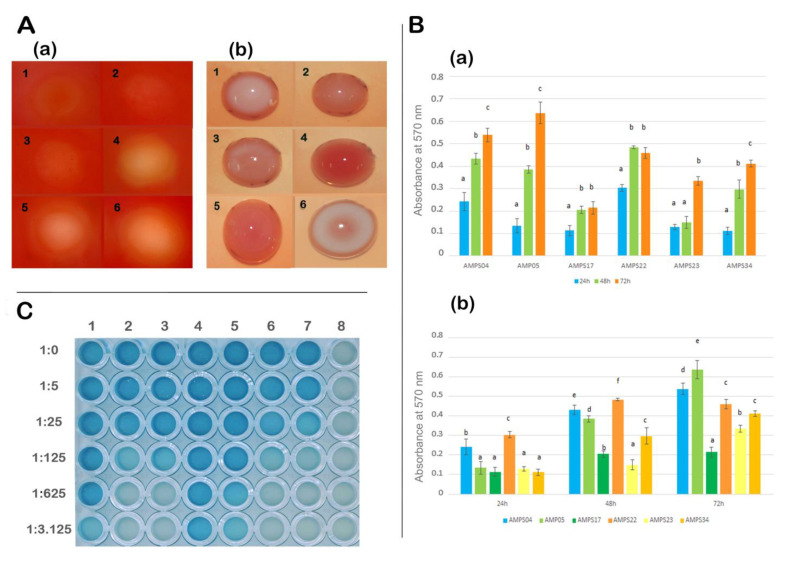
(Panel **A**): Cellulase production on CMC (carboxy-methyl-cellulose) (**a**) and cellulose-like polysaccharides production on plates containing Congo Red (**b**) by the strains: 1. AMPS04, 2. AMPS05, 3. AMPS17, 4. AMPS22, 5. AMPS23, 6. AMPS34. (Panel **B**): Absorbance values at 570 nm OD of CV-stained biofilms formed on PVC plates by the analyzed strains at different incubation times (**a**) and evolution along the time of these values for each strain (**b**). Each graph bar represents the average of at least eight wells. Error bars indicate the standard deviation. Values followed by the same letter in each treatment are not significantly different from each other at *p* = 0.05 according to Fisher’s Protected LSD test (least significant differences). (Panel **C**): Results of the bioassay production of *quorum sensing* molecules after serial dilutions of supernatants from liquid cultures of strains dispensed over a semisolid culture of the bioreporter strain *Agrobacterium tumefaciens* NTL4: 1. Positive control with 10 ng/mL of OHL (N-3-octanoyl homoserine lactone), 2. AMPS04, 3. AMPS05, 4. AMPS17, 5. AMPS20, 6. AMPS22, 7. AMPS34, and 8. Negative control with distilled water.

Cellulases and cellulose produced by *Rhizobium* strains are involved in plant colonization and biofilm formation in legume roots [36], and they are produced by several *Rhizobium* species that are able to form biofilms [36], including some strains from *R. laguerreae* isolated from *P. vulgaris* nodules [37]. In agreement with these results, all strains analyzed in this study produced cellulases and cellulose (Table 1, Figure 5A). They were able to form biofilms on abiotic surfaces, and an increase in the biofilm formation over time was found in all selected strains, with significant differences between the biofilm formation after 24 and 48 h, except in the strain AMPS23. Significant differences were also found after 72 h in strains AMPS04, AMPS05, AMPS23, and AMPS34 (Figure 5(Ba)). The strain AMPS05 exhibits the highest biofilm formation at 72 h and the strain AMPS22 at 24 and 48 h (Figure 5(Bb)). These results are in agreement with those found for *Rhizobium* strains nodulating clover [36] or common bean [37].

Rhizobial strains from populations formed on the legume roots communicate among them through signaling systems based on the quorum sensing compounds of the N-acyl-homoserine lactone groups (AHLs), which stimulate the genetic exchange among bacteria in the legume rhizosphere and play a direct role in the legume nodulation process [38,39]. Symbiotic rhizobia have different AHL-based quorum sensing systems, and within the genus *Rhizobium*, they have been studied in *R. etli* and *R. leguminosarum* [39]. In this study, we detected quorum sensing signals in culture supernatants of all strains, although some differences in active molecule production were found (Table 1, Figure 5C). Strains AMPS17 and AMPS22 were able to produce the highest estimated concentration of AHL, followed by strains AMPS04 and AMPS05, strains AMPS23 and AMPS34 being those producing the lowest AHL concentrations (Figure 5C). Differences in the production of AHL have been found in different strains and symbiovars of *R. leguminosarum* [40], but this is the first work in which the AHL production is compared in different *Rhizobium* strains isolated from legume nodules using the micro method described in the work of Trovato et al. [27].

### 2.6. In Vitro Plant Growth Promotion Mechanisms

The ability to fix nitrogen in symbiosis with legumes was the first plant growth promotion mechanism studied in members of the genus *Rhizobium* [41], but other mechanisms have been more recently reported, such as the ability to solubilize phosphate, to synthesize indole acetic acid, or to produce siderophores [42]. In this work, we analyzed these mechanisms in selected strains from each RAPD group showing that all of them produced IAA and siderophores, but only two of them were able to solubilize phosphate (Table 1). Strains AMPS04, AMPS05, and AMPS34 produced higher IAA amounts, which were similar or higher than those reported for other *Rhizobium* strains [37,43,44]. Concerning siderophore production, the strain AMPS34 produced the halo with the largest radius in the CAS medium, but in general the results were similar to those previously found for other *Rhizobium* strains [37,43,44]. The ability to solubilize phosphate was found in only two strains, AMPS23 and AMPS34 (Table 1), which agrees with previous works where this mechanism was less frequently found in *Rhizobium* strains [43]. All these results are in agreement with those obtained for other *Rhizobium* strains [43,44], including two *R. laguerreae* strains isolated from common bean and clover nodules [37,45].

### 2.7. Effectiveness on Pea Plants

The effectiveness of rhizobia-legume symbiosis is directly related to the symbiotic nitrogen fixation process carried out by the rhizobia inside the nodules [46]. Therefore, the nodulation ability is also directly related to the effectiveness of rhizobial strains, since nodule formation and growth are regulated in response to the N present in the legume phloem and/or in the soil [47,48]. Moreover, as previously indicated, symbiotic nitrogen fixation was the first studied mechanism of plant growth promotion; therefore, classically, the parameters analyzed to determine the effectiveness of rhizobial strains have been the number of nodules, the N content, and the fresh and dry matter of the legume shoots. The obtained results for these parameters in *P. sativum* plants inoculated with selected strains from this study are displayed in Table 2. They show that, as expected, all strains were able to nodulate *P. sativum*, and significant differences in the number of nodules per plant were found between the strain AMPS04 and the type strain of *R. pisi*. All inoculation treatments yielded *P. sativum* plants with significantly higher fresh and dry weights than those from the uninoculated control without nitrogen addition. Both parameters were significantly lower in all treatments, including the positive control plants inoculated with the type strain of *R. pisi*, with respect to the positive noninoculated control plants watered with a nutrient solution containing 0.5g/L of ammonium nitrate as N source. Among the inoculation treatments, those performed with the type strain of *R. pisi* and with the strain AMPS34 yielded higher values for fresh and dry weights. Both values were not significantly different between these treatments and with respect to those corresponding to the inoculation with the strains AMPS05 and AMPS17. Concerning the percentage of fixed N, all treatments presented significant differences with respect to the uninoculated control without added N, and no significant differences were found among the remaining treatments. The total N content per plant showed the same behavior as that of the shoot dry weight (Table 2). These results imply that, although some strains were more effective than others, in general the strains of *R. laguerreae* sv. viciae showed high symbiotic efficiency in pea plants, with no significant differences between plants inoculated with the type strain of *R. pisi* and those inoculated with the strain AMPS34. In this last case, the percentage of fixed N per plant was only 2.5% lower than in the plants watered with a nutrient solution containing N sources. These results imply that *R. laguerreae* strains established highly effective symbiosis with *P. sativum* and can be used as inoculants for this legume. 

## 3. Materials and Methods 

### 3.1. Diversity Analysis of Strains by RAPD Fingerprinting 

The strains were isolated in a previous work from effective nodules (pink colour) of *P. sativum* plants at the flowering stage in Aldea de San Miguel (41°27′37″ N 4°37′01″ W), located in Valladolid province (Spain), where this legume is commonly cultivated [12]. 

The genetic diversity of isolated strains was assessed by RAPD fingerprinting performed as previously described [50] with the primer M13 (5′- GAGGGTGGCGGTTCT–3′) and the Dream-Taq™ DNA Green PCR Master Mix (Fisher Scientific, Waltham, MA, USA). PCR conditions were the following: preheating at 95 °C for 9 min, 35 cycles of denaturing at 95 °C for 1 min, annealing at 45 °C for 1 min, and extension at 75 °C for 2 min, with a final extension at 72 °C for 7 min. Aliquots of 17 µL of each PCR product were electrophoresed on 1.5% (w/v) agarose gel in TBE buffer (100 mM Tris, 83 mM boric acid, 1 mM EDTA, pH 8.5) at 6 V/cm. The gels were stained in a solution containing 0.5 mg/L ethidium bromide, and photographed under UV light. Standard VI (Roche, Basilea, Switzerland) was used as a size marker. A dendrogram was constructed based on the matrix generated using the UPGMA method and Pearson’s coefficient with Bionumerics version 4.0 (Applied Maths, Austin, TX, USA).

### 3.2. Gene Sequencing and Analysis

The amplification and sequencing of *rrs*, *recA*, *atpD*, and *nodC* genes were carried out as indicated by Carro et al. [51], Gaunt et al. [23], and Laguerre et al. [33], respectively. These genes were sequenced at the Sequencing DNA Service (NUCLEUS) from Salamanca University (Salamanca, Spain). The obtained sequences were compared with those from GenBank using the BLASTN program [52]. Sequences were aligned using the ClustalX software [53]. The distances were calculated according to Kimura’s two-parameter model [54]. Phylogenetic trees were inferred using the neighbor-joining (NJ) analysis [55]. MEGA7 software [56] was used for all these analyses.

### 3.3. Quorum Sensing Signals and Biofilm Production

For the evaluation of the production of quorum sensing signals, strains were grown in 20 mL of YMB medium [57] for 48 h. Then 2 mL were transferred to 18 mL of YMB medium and were incubated for 24 h at 28 °C. The cultures were centrifuged 4 min at 4500× *g*, and the supernatants were filtered with a 0.22 µm pore filter. The strain *Agrobacterium tumefaciens* NTL4 (pZRL4) was employed as a reporter of AHL (acylated homoserine lactone) quorum sensing molecules. This strain was inoculated in 20 mL of liquid AB medium [58] supplemented with 30 µg/mL gentamycin and was incubated for 24 h at 28 °C. Afterwards, 10 mL of this culture were mixed with AB medium with agarose (1% *w*/*v*) at 43 °C and 150 µL of X-gal (5-bromo-4-chloro-3-indolyl-beta-D-galactopyranoside) stock solution for a final concentration of 60 µg/mL. Aliquots of 200 µL of this suspension were dispensed in each well of a 96-well microtiter plate. Upon medium solidification, 10 µL of a solution containing 10 ng/µL of OHL (N-Octanoyl-DL-homoserine lactone, Sigma Aldrich Co., St. Luis, MO, USA), filtered supernatants, and their serial dilutions were added. OHL was chosen as the AHL positive control. After 24 h of incubation, the digital image was acquired by an HP Scanjet 8200 flatbed scanner.

The ability of strains to form biofilms on abiotic surfaces was measured using the microtiter plate assay crystal violet post-staining as described by Robledo et al. [36]. The strains were grown in the minimal medium until reaching a value of 2.0 at 600 nm OD (ca. 1 × 10^7^ cells/mL). A decimal dilution was used for subsequent biofilm formation measurements [36]. Biofilm data were treated with one-way ANOVA and the Fisher’s post hoc test at a *p* value ≤ 0.05, using StatView 5.0 (Abacus Corporation, Berkeley, CA, USA). Data were analyzed by one-way ANOVA, and mean values were compared with Fisher’s protected LSD test (least significant differences) (*p* ≤ 0.05).

Cellulase production was tested on CMC double-layer plates as was previously described [59] and after 7 days the presence/absence of hydrolysis halos was recorded. Cellulose detection assay was carried out as described by Robledo et al. [36]. 

### 3.4. In Vitro Plant Growth Promoting Mechanisms

The solubilization of insoluble phosphate was analyzed on YED-P plates containing 2% CaHPO_4_ or Ca_3_(HPO_4_)_2_, which were incubated for 15 days at 28 °C [60]. Phosphate-solubilization effectiveness was calculated as the ratio between the halos around the colony with respect to colony size [28]. Siderophore production was evaluated in M9-CAS-AGAR [61] modified with the addition of a cationic solvent, HDTMA, which stabilizes the Fe-CAS complex and gives the medium its characteristic color [62]. Indole acetic acid production was evaluated in JMM medium [63] supplemented with 107 mg/L of tryptophan. After 7 days of incubation, the supernatants were recovered by centrifugation at 5000× *g* and filtered using 0.22 µm Millipore filters (Millipore Co., Burlington, MA, USA). Afterwards, 1 mL of Salkowsky reagent was added to 2 mL of supernatant, and the red color formed was measured by spectrophotometry at 550 nm using an ATI Unicam 8625 Spectrometer (Mattson, Madison, WI, USA) [64]. 

### 3.5. Effectiveness Assays in Pisum sativum 

For these assays, seeds of *Pisum sativum* var. “Rondo” were surface-sterilized with 5% sodium hypochlorite for 25 min. The sterilized seeds were germinated in sterile vermiculite and then inoculated with 1 mL per plant of a suspension containing approximately 1 × 10^8^ CFU/mL of each strain. Cells were obtained from cultures on YMA plates incubated for 5 days at 28 °C and were suspended in sterile water. The inoculated plants were placed in a plant growth chamber with mixed incandescent and fluorescent lighting (400 microeinsteins m^−2^ s^−1^; 400–700 nm), programmed for a 16 h photoperiod, day–night cycle, with a constant temperature varying from 25 to 27 °C, and 50–60% relative humidity. Plants were watered with nitrogen-free Rigaud and Puppo [49] nutrient solution. A positive control was watered weekly with the same solution, with 0.5 g/L ammonium nitrate. Plants inoculated with the type strain of *Rhizobium pisi* DSM 30132^T^ were included as a reference. Four weeks after inoculation, plants were harvested, the number of nodules was counted, the fresh and dry weight, and the nitrogen content of the aerial parts of plants were measured. This last analysis was performed in the Ionomic Service from CEBAS-CSIC (Murcia, Spain). Data were analyzed by one-way ANOVA, and mean values were compared by Fisher’s protected LSD test (least significant differences) (*p* ≤ 0.05).

## 4. Conclusions

Collectively, the results of this study showed the following: (i) although several type strains of different species from the group of *R. leguminosarum* have identical *rrs* gene sequences, some differences have been found in this gene with respect to other strains of these species, including the presence of an insert (IVS) of 75 bp at the beginning of the *rrs* gene. (ii) Strains carrying identical IVS in their *rrs* genes were isolated from nodules of *Pisum*, *Lens*, and *Vicia* in different countries from Europe and Asia. (iii) All these strains belong to the species *R. laguerreae* according to the results of the *recA* and *atpD* gene analysis, which suggests that several of these strains are currently misnamed in GenBank. (iv) All strains analyzed in this study have been identified as *R. laguerreae* after *recA* and *atpD* gene analysis, and they belong to different clusters within this species, whose complexity was confirmed in this work. (v) Further studies based on whole genome analysis are necessary to establish cut-off values for genospecies or subspecies differentiation as well as to propose the correction of the misnamed strains. (vi) *R. laguerreae* is widely distributed in nodules of *Pisum*, *Vicia*, and *Lens*, three grain legumes cultivated for human and animal feeding worldwide, but some strains assigned to this species presented similarity values in their *recA* and *atpD* genes lower than those currently accepted for species differentiation. (vii) The analysis of the *nodC* gene showed that all strains analyzed in this study belong to the symbiovar viciae, which is distributed worldwide in the nodules of legumes from *Pisum*, *Lens*, and *Vicia*. (viii) This symbiovar is phylogenetically very complex and currently encompasses strains showing *nodC* gene similarity values lower than those presented by recently described symbiovars, evidencing that a consensus is necessary to establish cut-off values for symbiovar delineation. (ix) All strains from this study produced molecules involved in plant colonization and quorum sensing, such as cellulose, cellulases, and N-acyl-homoserine lactones. (x) These strains also produced IAA and siderophores, but the phosphate solubilization of tricalcium phosphate was only observed for two strains. (xi) All strains were effective on pea plants, with the strain AMPS34 as the most efficient, without significant differences with respect to the type strain of *R. pisi* used as a reference in this study. (xii) Taking into account the wide distribution of *R. laguerreae* sv. viciae strains in different countries and continents in *P. sativum* nodules and their high effectiveness in symbiosis with this legume, *R. laguerreae* sv. viciae is a promising candidate for the biofertilization of peas worldwide. 

## Figures and Tables

**Table 2:** Effectiveness of selected strains on *Pisum sativum* plants.

Treatment	Nodules/Plant (± S.E.)	Shoot Fresh Weight (g)/Plant (± S.E.)	Shoot Dry Weight (g)/Plant (± S.E.)	N (%) (± S.E.)	Total N (mg)/Plant (± S.E.)
Uninoculated plants (without N)		1.19 (±0.02) ^a^	0.21 (±0.02) ^a^	2.77 (±0.08) ^a^	5.72 (±0.13) ^a^
Uninoculated plants (with N) *		2.81 (±0.03) ^f^	0.45 (±0.03) ^f^	4.28 (±0.12) ^bc^	19.22 (±0.26) ^f^
*R. pisi* DSM 30132^T^	52 (±2.44) ^a^	2.74 (±0.04) ^e^	0.42 (±0.03) ^de^	4.15 (±0.09) ^bc^	17.49 (±0.29) ^e^
AMPS04	60 (±2.31) ^b^	2.59 (±0.03) ^bcd^	0.38 (±0.04) ^c^	4.10 (±0.10) ^b^	15.66 (±0.34) ^c^
AMPS05	52 (±2.74) ^a^	2.66 (±0.02) ^cde^	0.39 (±0.03) ^cd^	4.14 (±0.13) ^bc^	16.12 (±0.23) ^cd^
AMPS17	53 (±2.55) ^a^	2.66 (±0.03) ^cde^	0.40 (±0.03) ^d^	4.13 (±0.07) ^bc^	16.70 (±0.26) ^d^
AMPS22	55 (±2.14) ^ab^	2.55 (±0.03) ^bc^	0.36 (±0.05) ^b^	4.08 (±0.05) ^b^	14.72 (±0.28) ^b^
AMPS23	52 (±1.86) ^a^	2.65 (±0.02) ^cd^	0.39 (±0.03) ^cd^	4.14 (±0.11) ^bc^	15.94 (±0.27) ^cd^
AMPS34	51 (±1.79) ^a^	2.70 (±0.04) ^e^	0.42 (±0.04) ^de^	4.17 (±0.08) ^bc^	17.42 (±0.28) ^de^

Values followed by the same letter in each treatment are not significantly different from each other at *p* = 0.05 according to Fisher’s protected LSD test (least significant differences). S.E. = standard error. * Plants were watered with Rigaud and Pupo solution [49] containing 0.5 g/L of ammonium nitrate.
